# Is the timing of menarche correlated with mortality and fertility rates?

**DOI:** 10.1371/journal.pone.0215462

**Published:** 2019-04-18

**Authors:** Gabriel Šaffa, Anna Maria Kubicka, Martin Hromada, Karen Leslie Kramer

**Affiliations:** 1 Laboratory and Museum of Evolutionary Ecology, Department of Ecology, Faculty of Humanities and Natural Sciences, University of Presov, Prešov, Slovakia; 2 Department of Zoology, Faculty of Science, University of South Bohemia, České Budějovice, Czech Republic; 3 Department of Zoology, University of Life Sciences in Poznań, Poznań, Poland; 4 Faculty of Biological Sciences, University of Zielona Góra, Zielona Góra, Poland; 5 Department of Anthropology, University of Utah, Salt Lake City, Utah, United States of America; University of Botswana, BOTSWANA

## Abstract

Timing of menarche has largely been studied in the context of a secular trend. However, since mortality and fertility rates are fundamental demographic factors linked to a population’s developmental and reproductive characteristics, we expect that the timing of menarche, a precondition to reproduction, is also associated with these vital rates. We conduct an analysis of 89 countries and 21 demographic, socioeconomic, nutritional, and educational variables selected for their known influence on menarche. Model results predict that a country’s fertility and adult female mortality rates are significant predictors of mean age at menarche, while other covariates are not. Specifically, menarche is delayed in countries with high mortality and high fertility, which may be proxies for assessing overall environmental quality. We emphasize that, for a comprehensive understanding of the timing of menarche, it is critical to take into account both individual- and population-level influences.

## Introduction

Menarche is a key life history event shaping the onset of a female’s reproductive career [1–8]. Unlike other pubertal characteristics that manifest gradually (e.g. breast and pubic hair development, fat deposition, and the adolescent growth spurt), menarche is a discreet event [6,9]. As such, it is a useful, but underrepresented, variable in demographic research [7].

The timing of menarche has largely been studied in the context of a secular trend. Over the last century, a general decline in the age at menarche has been well documented [1,4,10–17]. Many drivers have been proposed to explain this global phenomenon [4,10,11,14,16–20]. While age at menarche clearly has a genetic component [1,21,22], earlier ages at its onset are attributed to a variety of lifestyle and socioeconomic factors, including residence in a rural or urban setting, country economics (e.g. Gross Domestic Product (GDP)), family income, parental or sibling education, family size or exposure to environmental chemicals [23,24]. Nutritional and health factors that have been the focus of much research include energy intake, body mass index (BMI), consumption of particular macronutrients such as sugar or fat [25–28] or alcohol [29]; for a detailed overview of covariates and studies, see S1 Table.

While a breadth of factors likely contribute to variation in menarcheal age, most research has focused on individual responses. However, population characteristics also influence individuals [30–32]. For example, expectations about local mortality have been shown to shape fertility decisions [33–36]. In particular, among American and Canadian study subjects, an earlier onset of reproduction was associated with low neighborhood life expectancy [37] and perceptions of environmental uncertainty were shown to affect reproductive timing among low-income African American female teenagers [33]. In experimental studies, mortality perceptions influenced fertility preferences of student participants [38,39]. Thus, individuals appear to adjust their developmental and reproductive timing to their perception of future survival prospects in relation to local life expectancy and/or mortality rates [35–37]. As a consequence, this affects the average expression of the strategy within a population [40,41]. This suggests that a bidirectional relationship exists between individual responses and population-level characteristics [32]. Following on this, we suspect that mortality and fertility rates can be used as proxies for overall environmental quality and resource availability to which individuals respond in an adaptive way [33,36,37]. Here we consider population-level characteristics as an unexplored approach to the timing of menarche.

Mortality and fertility rates are fundamental demographic factors [42] that influence organisms’ life history [40,43,44]. As examples, Charnov and Berrigan [44] developed a mathematical model in which mortality rates across a range of mammalian taxa effectively predict growth and fertility rates. Promislow and Harvey [40] showed that mortality is the best predictor of variation in life history characteristics across various mammalian species. In humans, many within- and between-population studies have shown that mortality schedules influence differences in growth rates and reproductive strategies [5,36,41,43,45–49]. However, the association between mortality and fertility and the timing of menarche has received much less attention. As an exception, Walker et al. [49] found that in some small-scale societies, higher mortality rate is associated with faster development, earlier menarche, and age at first reproduction.

Moreover, research on menarcheal timing [4,49–52] has focused predominantly on a particular country, region, ethnic group or different kinds of subjects within populations, such as city residents, students or clinical patients [6,15,20,49,53–63]. Such research often has been conducted in high-income Western countries, which represent an exceptional and recent phenomenon contributing to human developmental and socio-cultural variation [64,65]. Despite great progress in understanding the causes of the timing of menarche at the individual level, population-level processes underlying global variation in menarcheal age remain understudied.

**Fig 1:**
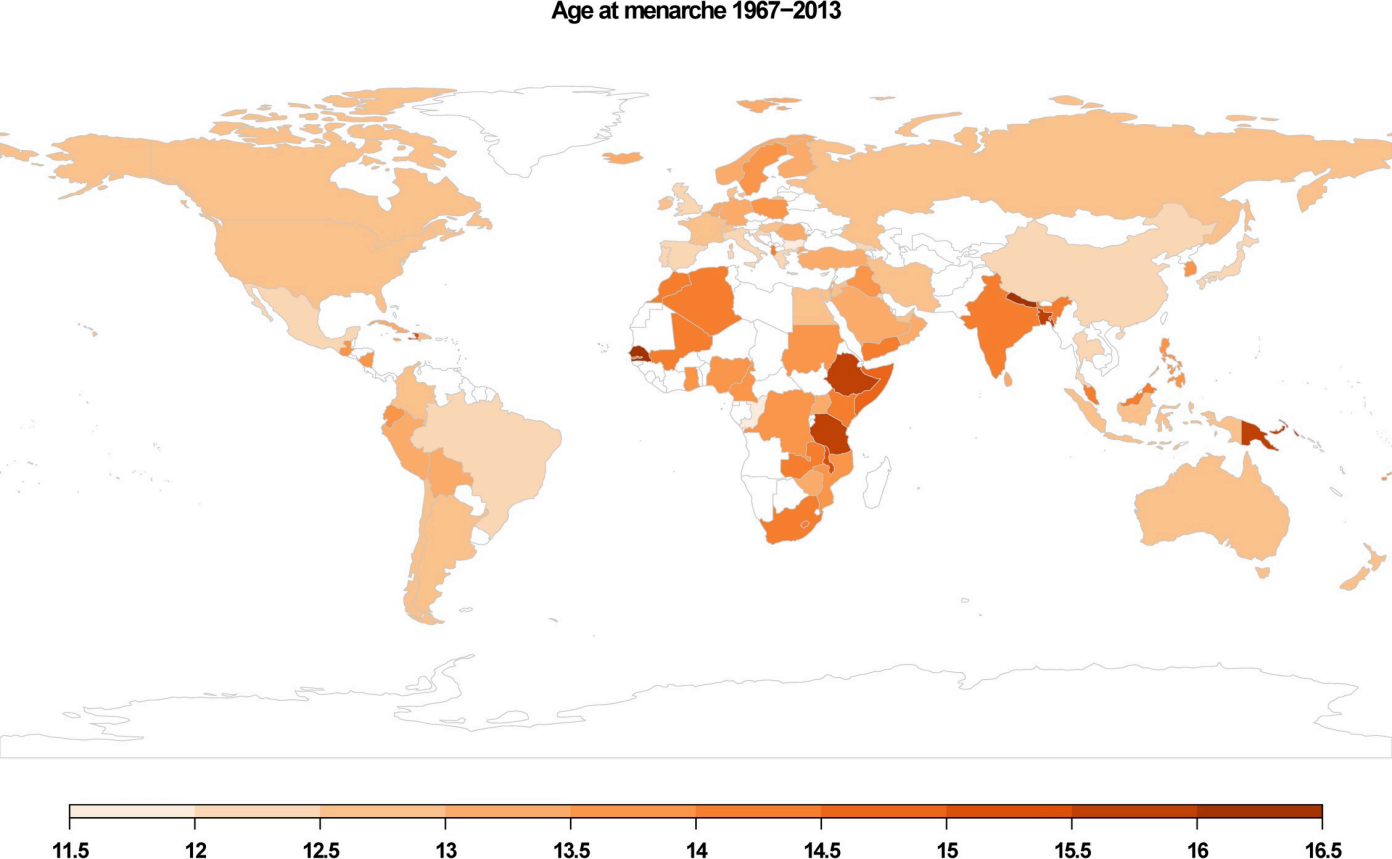
Global distribution of the dependent covariate mean age at menarche for 89 countries included in the sample. Each country is plotted by mean age at menarche from a given year. Legend shows scaled ages at menarche.

To fill this gap, this study investigates the timing of menarche using a comprehensive, global cross-country dataset (Fig 1) and explores the relationship between population-level characteristics and age at menarche. As far as we know, this is the only published compilation of such data. We predict that both a country’s 1) mortality rate and 2) fertility rate will influence mean age at menarche.

## Material and methods

### Data collection

We assembled a database for 89 countries with mean menarcheal age data and 21 demographic, socioeconomic, nutritional, and educational covariates (Table 1 and S2 Table). The sample of countries was determined by the availability of menarcheal data, which were drawn from published sources. However, since no global database lists menarcheal age as a demographic variable [7], mean age at menarche for a country was established by the authors from previously published studies for a particular country or for a group of countries (e.g. Eveleth and Tanner [11]; Thomas et al. [52]). Included here are known menarcheal studies published between 1967 and 2013 (S2 Table).

**Table 1:** Definitions and sources of the analysed covariates.

Covariate	Definition	Reference
**Demographic**		
Mean age at menarche	age of first menstruation	various sources (see S2 Table)
Life expectancy at birth	total (years)	[66]
Fertility rate	total (children per woman)	[66]
Adolescent fertility (15–19)	births per 1,000 women aged 15–19	[66,67]
Maternal mortality ratio	modelled estimate, per 100,000 live births	[66,67]
Infant mortality	per 1,000 live births	[66]
Under-five mortality	per 1,000 live births	[66]
Adult female mortality	probability of female dying between the ages 15–60	[67]
Adult male mortality	probability of male dying between the ages 15–60	[67]
Total adult mortality	probability of dying between the ages 15–60	average adult male/female mortality rate
**Socioeconomic**		
Average size of household	average number of persons per household	various sources (see S1 Dataset)
Rural population	% of total population	[67]
Energy use	kg of oil	[67]
Fossil fuel energy consumption	% of total energy consumption	[67]
Gross domestic product (GDP) per capita	current US$	[66]
Livestock production index	meat, milk, dairy, eggs, etc.	[67]
Food production index	food crops (except coffee and tea)	[67]
**Nutritional**		
Energy consumption per capita	kcal/person/day	various sources (see S1 Dataset)
Sugar consumption	g/person/day	[66]
Body mass index (BMI) female	kg/m^2^	[66]
**Educational**		
Out of primary school, female	number of females not enrolled in either primary or secondary schools	[66]
Primary completion rate, female	% of females entrants in the last grade of primary education	[66]

While we have conducted a thorough literature search and made the most of the global mean menarcheal age data available, the source studies on menarcheal age included in our sample (S2 Table) may not be representative of the country as a whole, and in most cases (72 out of 89) are necessarily derived from sub-populations specified by region, ethnic group or various socio-economic strata. Studies were considered nationally representative (17 out of 89) only when 1) authors stated it explicitly, 2) their data were derived from nation-wide surveys or 3) the data sampling locations chosen by authors covered roughly a country’s area (see S2 Table). Additionally, if more than one record for menarcheal age in a study was available for a country (Belgium, Brazil, Cameroon, China, Iraq, Jordan, Singapore, Sudan, and Uganda), an average was calculated. In case of two countries, Albania and United Kingdom, given years for the age at menarche in the sample represent average values calculated from time intervals (2000–05 in case of Albania and 1990–93 for the UK) over which data in both studies were collected. Mean menarcheal age for Belgium was taken from the study of Flemish girls and mean menarcheal age for Uganda reflects post-conflict state. However, in the latter study, authors report no effect of such stressful events on menarcheal age in their sample.

We selected 21 demographic, socioeconomic, nutritional, and educational covariates based on their demonstrated influence on the timing of menarche (Table 1 and S1 Table). Most covariates were available for most countries, and the year in which the covariate data were collected was matched as closely as possible to the year in which menarche data were collected or the study was published. When we were unable to do so, we used information gathered within five years *prior* to the reference year for menarcheal age, since the timing of menarche would be sensitive to prior not subsequent conditions. However, because data for average household size (N = 70), out of primary school (N = 56), and primary completion rate (N = 55) are lacking for many countries, we drew from a broader temporal period and employed an interval of ±5years. Reference years for average size of household for Norway and Singapore represent average values calculated from the two periods. The list of tested covariates with definitions and references is included in Table 1.

### Data caveats

An ideal dataset to evaluate a causal relationship between population-level characteristics and age at menarche would have matched menarcheal data with regional populational traits collected at the same time period across multiple regions. To our knowledge, such data do not exist. While we do not have individual-level characteristics to link to menarcheal age, covariates added to the analysis here represent indicators of the general (e.g. nutritional, socio-economic, demographic) status of the population in which girls are maturing. Unfortunately, aggregate data and covariates chosen do not necessarily capture sufficient information regarding childhood and adolescence. We do feel, however, that we have carefully made the most of the age at menarche data available for a study that is mostly of explorative character. Because we are interested in age at menarche as an understudied but critical and discreet life history marker that is theoretically predicted to be sensitive to environmental circumstances, our goal is to first evaluate if such an association emerges between background mortality and fertility conditions. The results of this research will hopefully then generate predictions that can be evaluated with local, and individual level data.

### Statistical procedure

The 21 covariates and the outcome variable (mean age at menarche) were tested for normality using the Shapiro-Wilk’s test and for homogeneity of variances using the Levene’s test (statistically significant at p<0.05). Covariates that did not fulfil the normality criterion were log- or square root- transformed and then tested for normality again. Since only two covariates were normally distributed (female BMI and sugar consumption), non-parametric tests were employed (S3 Table). Descriptive statistics of the sample therefore include measures for non-normally distributed data, such as inter-quartile range (IQR), median, and min. and max. values (S3 Table). To account for a possible spatial autocorrelation between countries due to similar environmental (e.g. climate) or economic (e.g. migration, type of economy) factors, we evaluated whether the observed value of a covariate in one country significantly depended on the value for the same covariate in neighbouring countries. We used centroids for each country to quantify the spatial autocorrelation using Mantel’s I coefficient for each variable’s class distance (S4 Table).

To obtain a general view of the data, associations between mean age at menarche and the 21 covariates were calculated using the Rho-Spearman’s rank correlation coefficients (p<0.05) (S5 Table). To select a refined set of covariates for a GLM model construction, we used a Partial Least Squares (PLS) regression with the mean age at menarche as a dependent covariate. Covariates with the highest values were selected (p<0.05). As we expected collinearity to occur among population-level covariates, we used PLS instead of a Prinicpal Component Analysis (PCA) because it is more sensitive test of autocorrelation. Moreover, because the full suite of covariates is not available for all countries (only 6 variables were available for all 89 countries), PLS is better suited to accomodate missing data than PCA.

Having selected a set of covariates with the highest value of standardised coefficient in PLS regression (Table 2), we used GLM procedure to model the probability that mean age at menarche is a function of these predictor variables. GLM is appropriate in this case because it allows both dependent and independent response covariates to have a non-normal distribution. We used a stepwise (backward elimination) procedure and based our model selection on Akaike information criterion (AIC) scores. This index measures the quality of statistical models in an absolute sense by giving a relative estimate of the information lost when trading-off the goodness-of-fit and complexity (i.e. number of predictors) of the model. Therefore, the best-fit model was characterised by the lowest AIC value.

**Table 2:** Standardised coefficients in the PLS regression.

Covariate	N	Standardized model coefficients
**Demographic**		
Life expectancy at birth	89	-5.224*
Fertility rate	89	0.972*
Adolescent fertility (15–19)	89	-0.432
Maternal mortality ratio	73	-0.767
Infant mortality	87	-4.438*
Under-five mortality	87	4.069*
Adult female mortality	87	0.877*
Adult male mortality	87	-0.899*
Total adult mortality	87	-0.014
**Socioeconomic**		
Average size of household	70	-0.607
Rural population	89	-0.065
Energy use	79	-0.607
Fossil fuel energy consumption	78	-0.402
Gross domestic product (GDP) per capita	84	-0.572
Livestock production index	89	-0.101
Food production index	89	-0.144
**Nutritional**		
Energy consumption per capita	72	0.021
Sugar consumption	75	0.014
Body mass index (BMI) female	79	0.021
**Educational**		
Out of primary school female	56	0.316
Primary completion rate female	55	-0.470

Asterisk (*) indicates covariates selected for GLM analysis.

**Fig 2:**
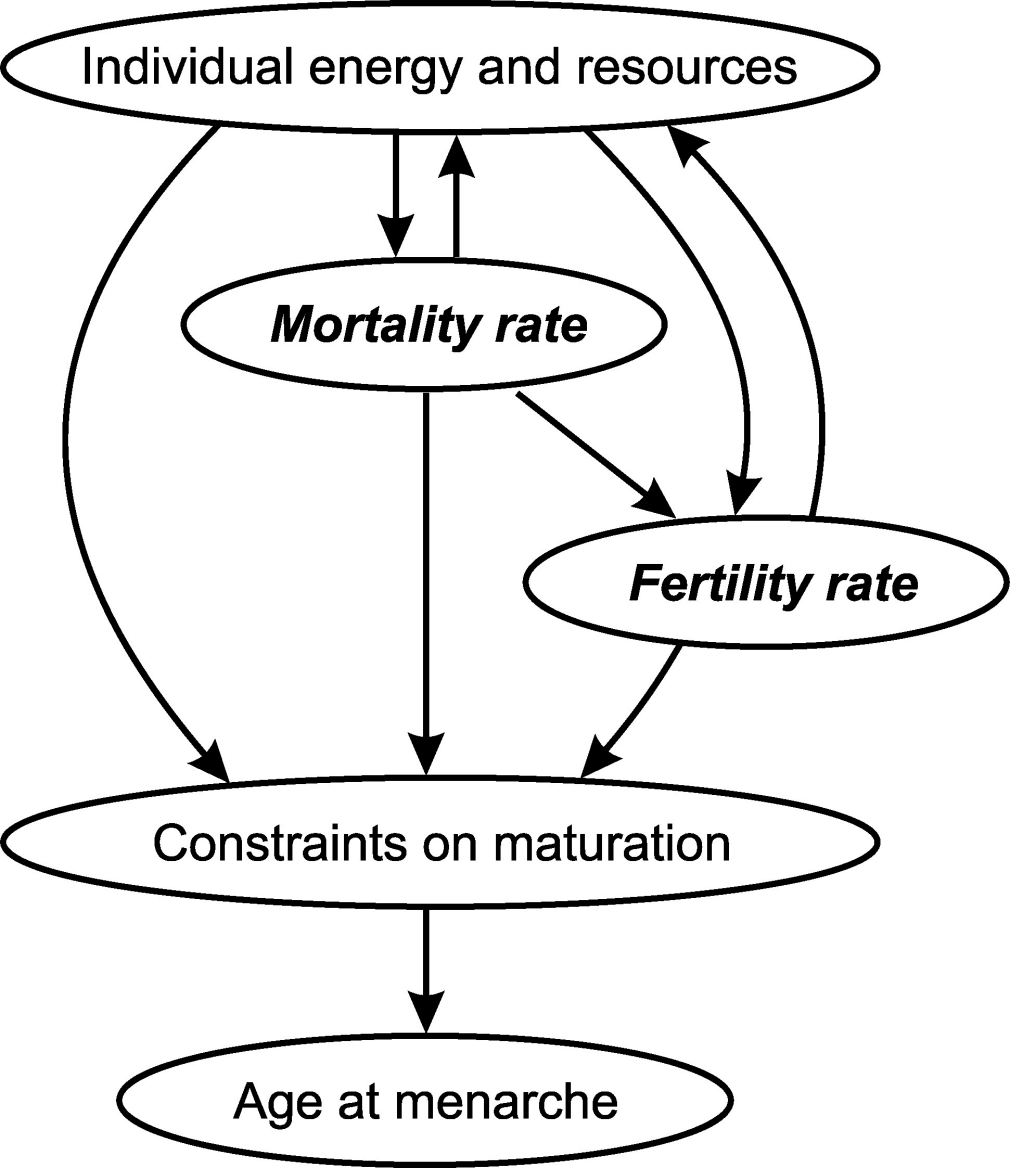
Graphical representation of the main findings. Arrows depict bidirectional associations of individual- and population-level covariates with the timing of menarche. Circles filled with italic and bold point to the model results.

The descriptive statistics, spatial autocorrelation, GLM, and figure graphics were carried out in R software (version 3.5.1) [68] with *ade4* package [69] for spatial autocorrelation test and *rworldmap* package [70] and *DiagrammeR* package for creating Figs 1 and 2, respectively. The Rho-Spearman correlation and PLS analyses were computed with SPSS software (version 22.0.0.1).

## Results

### Descriptive results

Univariate statistics including Interquartile range (IQR), median, min. and max. values for non-normally distributed data are summarized in S3 Table. The Mantel’s test for spatial autocorrelation between countries was non-significant for all 22 computed coefficients (including mean age at menarche), indicating no evidence for spatial autocorrelation (S4 Table). As expected, the Rho-Spearman correlation revealed that all demographic covariates, except life expectancy at birth correlating negatively (N = 89, SC = -0.681, p<0.001), are significantly positively correlated with mean age at menarche (S5 Table). In other words, menarche tends to occur later in countries with high rates of mortality and fertility and low life expectancy at birth (various measures; see Table 1). Except average household size (N = 70, SC = 0.453, p<0.001) and rural population (N = 89, SC = 0.652, p<0.001) correlating positively, all other socioeconomic and all nutritional factors were significantly negatively correlated with the age at menarche (S5 Table). For instance, earlier menarche is associated with countries with smaller families, higher percentage of urban populations, and/or higher GDP and energy consumption per capita and *vice versa*. The educational variables, female out of primary school and female primary completion rates, were positively (N = 56, SC = 0.596, p<0.001) and negatively (N = 55, SC = -0.532, p<0.001) significantly associated with mean age at menarche, respectively (S5 Table), suggesting that higher the degree of attained education, the earlier age at menarche.

### Model results

Based on the PLS analysis, the following six covariates with the highest scores were selected as predictors for GLM: life expectancy at birth, fertility rate, infant mortality, under-five mortality, adult female mortality, and adult male mortality (Tables 2 and 3). The stepwise GLM procedure showed that the model containing mean age at menarche as a dependent covariate and fertility rate (SE = 0.074, t-value = 2.651, p<0.009) and adult female mortality rate (SE = 0.001, t-value = 2.209, p<0.029) as predictor covariates explained the most of the variation in menarcheal age (variation explained is 41.38%; Table 3). The best-fit GLM model was characterised by the lowest AIC score (203.81). Model estimates and *p* values show that fertility rate has a greater influence on the mean age at menarche than adult female mortality rate.

**Table 3:** General linear model of the effects of fertility rate and adult female mortality on the mean age at menarche calculated for 89 countries.

Covariate	Estimate	Standard error	t-value	p-value
Intercept	12.277	0.181	67.673	<0.001
Fertility rate	0.196	0.074	2.651	0.009
Adult female mortality	0.003	0.001	2.209	0.029

AIC = 203.81

## Discussion

The results reveal two major findings. First, of the 21 covariates, fertility rate (children per woman) and adult female mortality rate (the probability of female dying between the ages 15–60) are the best predictors of variation in the timing of menarche across countries in our sample (Table 3). Although improvements in socioeconomic, health, and nutritional conditions are factors responsible for a shift to an earlier age at menarche at the individual level, our model results show that at the population level, the timing of menarche is strongly associated with fertility and mortality rates. Second, fertility (N = 89, SC = 0.681, p<0.001) and adult female mortality rates (N = 89, SC = 0.685, p<0.001) correlate positively with menarche (S5 Table), indicating that both high fertility and high mortality are associated with *later* menarche. As we argue below, our results are not mutually exclusive with previous findings but offer a perspective on how factors at the individual level can be translated into and viewed from the population level and *vice versa*. We discuss our findings in light of life history theory.

Life history theory explains variation across species in the timing and energy allocated to life events [34]. It posits that because an organism’s time and resources are limited, trade-offs are made whether to invest in maintenance (basic somatic functions to survive) versus growth or reproduction [43,44]. Selection pressures from mortality and fertility significantly influence a specie’s and a population’s life history strategies [40,43,44,47,49]. In particular, average age at first birth has been the focus of many theoretical and empirical studies. Because menarche is an important gateway event that initiates reproductive potential, background (i.e. population-level) mortality and fertility rates, although understudied, likely impact variation in its timing as well.

With respect to mortality, while a particular life history strategy may be optimized through energy or time allocation trade-offs, extrinsic mortality–an unavoidable source of mortality not under the control of an organism shared by all individuals in the population–persists [43,49,68,69]. Life history theory predicts that individuals will respond to high mortality environments by adjusting their developmental and reproductive strategies. At the level of species, high mortality is associated with faster growth, earlier maturity and reproduction, and shorter lifespans [40,43,44]. Low mortality, on the other hand, is associated with greater somatic investment, slower growth and maturation, longer lifespan and thus later reproductive competence [40,43,44,70].

Expectations about the relationship between mortality and other life history characteristics have been applied to both individual allocation decisions and to within- and between-populational differenes [41,43,44,71,72]. Age at menarche within and between populations varies greatly across time and space [11,41], and is correlated with many genetic, biological, and environmental variables. While we know that age at menarche is influenced by a constellation of individual factors, mortality rate also may be an important predictor of a population’s mean age at menarche.

However, the few comparable studies investigating the effect of various mortality rates on the timing of menarche show mixed results. For example, in some small-scale societies, such as Pumé and Hiwi of Venezuela, who live in high mortality environments, menarche occurs early [49,73–75], a finding consistent with Charnov’s life history perspective. Similarly, low life expectancy, which is usually correlated with high infant mortality, is associated with early menarche [35,37]. In studies using aggregated data, Danker-Hopfe [51] found no association between either life expectancy or infant mortality and age at menarche in a sample of 19 European countries. On the other hand, Thomas et al. [52] found in the sample of 67 countries that low life expectancy predicts later menarche. Ellison [50] reports similar findings; in a sample of 37 human populations, high infant mortality positively correlates with later menarche.

Although our results likewise demonstrate a strong relationship between mortality rate and mean age at menarche, it is not in the theoretically expected direction. Rather, in our sample of countries, high mortality, and specifically adult female mortality, is associated with later menarche. This finding contrasts with Charnov’s prediction and other empirical studies [49,76–78], which show that high mortality rates are associated with faster maturity.

One possible explanation is that aggregated data can produce opposite associations to those at the individual level [30]. Another possible explanation why age at menarche is later under high-mortality conditions is becasue life history models assume that conditions are constant, and thus at equilibrium [79]. However, real, not predicted, living conditions can change substantially over a relatively short period of time which, coupled with dynamic socio-economic and political changes, particularly in developing countries, may cause that life history predictions do not necessarily hold [80]. For example, although Anderson [80] in the sample of sub-Saharan African countries found significant associations of life expectancy with other variables studied, the direction was the opposite of what should be expected from life history theory. It could also explain why we did not find evidence for spatial autocorrelation in our data spanning from 1967 to 2013. Although our results could suffer from these methodological limitations, there are a number of reasons why high mortality might be associated with later age at menarche at the population level.

In particular, girls growing up in challenging environments may adaptively assess their futures and adjust their maturation pace based on the population around them [33,37]. If others are dying, high mortality rates may function as a cue that conditions are not favourable and to mature more quickly. Thus, population characteristics, or rates, i.e. the probability of dying, may serve as signals of current and/or future conditions and life expectations to which an individual’s decisions are density dependent and based on what others are doing in the population [32,34,37].

Still, based on the nature of aggregated data, it is difficult to interpret why *adult female* mortality among other mortality measures (e.g. infant mortality) in the model (Tables 2 and 3), is the best predictor of mean age at menarche. It has been found, however, that in some natural fertility populations, i.e. populations that do not use parity-specific birth control, child’s nutritional status and survival prospects are, unlike patrilineal kin, dependent on the presence of a living mother and other matrilineal kin, such as maternal grandmothers and/or older sisters [81]. It is possible that adult female mortality rate may also reflect the presence/absence of matrilineal kin which could explain the association we found at the population level.

Finally, life history predictions are in specific reference to extrinsic mortality (see above). However, mortality can also be care- and resource- dependent [68,82], and risk individually variable, particularly if associated with nutrition and social resources, such as access to health care [36]. Under these circumstances, girls may be energetically constrained from early development (e.g. Eveleth and Tanner [11]). Poor environments characterized by low energy availability and high mortality may favor slower growth, later maturity and delayed menarche [49]. For example, while Hiwi (Venezuela), Pumé (Venezuela), and Baka (African pygmies, Cameroon) live in high mortality environments due to infectious diseases and parasites, they have fast life histories, including early menarche. In contrast, the Ju/’hoansi (Botswana/Namibia) and Gainj and Asai (Papua New Guinea), also live in high mortality conditions but mostly due to the risk of starvation and have slow development, later menarche, and later reproduction [49]. Thus, mortality rate may be an indirect proxy for current and/or future conditions to which individuals respond in an adaptive way adjusting their developmental and reproductive patterns [36]. How individuals adjust, and its effect on population means, may importantly depend on the level of resource-dependent mortality.

Mortality (defined as life expectancy, infant mortality or a general rate) thus appears 1) to be an important predictor of the age at menarche both at the population and individual level (but see Kyweluk et al. [64]), and 2) to be variably positive or negative. We caution against deriving any causal inferences and simple interpretations that country-level data predict a positive relationship between mortality and age at menarche, while individual-level data predict a negative relationship. Rather both might be the case, depending on the nature and/or source of mortality.

Fertility rate is another strong predictor of menarcheal age (Table 3), with menarche occurring later in high-fertility countries. Following on resource-dependent mortality, one interpretation of this finding may be that high fertility increases energetic demands on mothers to provide sufficient resources for dependent offspring, which may result in competition for resources among siblings and with adults [83]. For example, in a longitudinal study using aggregated data for rural communities of Bangladesh, declining fertility rate led to significantly lower child mortality rates [84]. Presence of younger sibling/s in the household increases competition for food and other family resources. At the population level, lower fertility means more public resources available per each offspring [84]. This is particularly true in market-based economies, in contrast to traditional labor-based economies where the number of children is associated with increased household production and greater wealth [85]. If, however, energy consumption per offspring decreases with increasing fertility, it may result in slower growth and later ages at menarche [86] (but see Kramer et al. [85]). We also report significant negative correlations between our nutritional covariates and age at menarche (S5 Table).

Individual-level studies on the relationship between menarche and both family size and number of siblings may support this assertion [1,20,54,62,87,88]. For instance, family size and birth order had the strongest effect on menarcheal age in the sample of Portuguese university students with menarche occurring later with increasing number of children in the family [89]. In another study of three Polish provinces (Kraków, Opole, and Nowy Sącz) that differed in the degree of urbanization, family size was among the most significant socioeconomic variables predicting menarcheal age [54]. Particularly, in the least urbanized province, Nowy Sącz (less than 35% of population live in cities), family size was the only significant predictor of menarcheal age [54]. It is likely that pre-reproductive girls allocate substantial amount of resources to a direct care of their younger siblings, which, resulting in increased energy expenditure of girls, and eventually decreased energy consumption per capita, could be mirrored in later ages at menarche.

Taken together, the timing of menarche is likely a result of an optimization of trade-offs resulting from energetic constraints on growth and mortality pressure for an onset of fertility (Fig 2). An example of such a case could be seen in low mortality/low fertility countries, in which, unlike in high mortality/high fertility countries, sufficient amounts of energy drive menarche into earlier ages, but age at first reproduction is, perhaps due to (perceived or real) predictability or stability of the environment and resources postponed into later ages. Future research could focus on exploring the interactions, in terms of a length of an interval, between the timing of menarche and age at first reproduction along the dimensions of mortality and fertility within the context of demographic transition.

## Conclusions

This study investigated population-level influences on the timing of menarche. We found that (adult female) mortality and fertility rates have significant effect on the variation in mean menarcheal age across countries in our sample, which is consistent with general life history framework. It is clear that the energetic and social resources available to an individual have a direct influence on the timing of menarche. At the population level, if this is reflected in mortality and fertility rates, the latter may act as proxies for assessing overall quality of the environment. This, in turn, can exert their indirect effects on the individual timing of menarche (Fig 2). Thus, for a comprehensive understanding of the timing of menarche it is critical to take into account both individual- and population-level influences. We assert that a population-level age at menarche can be another important demographic variable.

## Supporting information

S1 Table Overview of the most commonly associated covariates with mean menarcheal age in the literature. (DOCX)

S2 Table Mean age at menarche for 89 countries with year and reference. (DOCX)

S3 Table Univariate statistics for non-normally distributed data with IQR, median, min and max values for the covariates included in the analyses. (DOCX)

S4 Table Analysis of spatial autocorrelation (Mantel’s coefficient) between mean age at menarche and the remaining covariates. (DOCX)

S5 Table Rho-Spearman correlation of the tested covariates with the mean age at menarche. (DOCX)

S1 Dataset (XLS)
