# Supplementary material for: Is the timing of menarche correlated with mortality and fertility rates?
Source: PLoS One. 2019 Apr 18;14(4):e0215462. doi: 10.1371/journal.pone.0215462 (PMC6472797; doi:10.1371/journal.pone.0215462)
Supplement: S1 Table — (DOCX) [file pone.0215462.s001.docx]

**S1 Table. Overview of the most commonly associated covariates with mean menarcheal age in the literature.**

| Covariate | Reference |
| --- | --- |
| Demographic | |
| Life expectancy* | [1–4] |
| Fertility rate* | [1] |
| Adolescent fertility (15-19)* | [5] |
| Mortality rate – infant* | [3,6] |
| Mortality (total, all-cause, adult)* | [7–9]** |
| Socioeconomic | |
| Household/Family size (number of siblings)* | [10–23] |
| Rural population (%)* | [12,20–22,24–29] |
| Gross domestic product (GDP)/Gross national product (GNP)* | [1,3,30] |
| Family income | [2,12,15,19,31,32] |
| (Mother’s) employment | [19] |
| Nutritional | |
| Energy intake* | [1,3,31,33–40] |
| Sugar consumption (g/person/day)* | [41,42] |
| Carbohydrate (%) | [36–38,41,43,44] |
| Protein (g/person/day) | [3,31,33,36–38,41,43] |
| Fat (g/person/day) | [3,33,36–38,40,41] |
| Prevalence of undernourishement | [28,45,46] |
| Body mass index (BMI)* | [9,19,29,33,40,47–52] |
| Prevalence of overweight | [50,53] |
| Prevalence of diseases/infections | [39] |
| Chemicals | [54,55] |
| Physical activity/Energy balance | [1,40,45,56] |
| Educational | |
| Adult illiteracy rate | [1,57] |
| Education/School enrollment (parents or children) | [2,12,59–61,19–23,31,52,58] |

*Covariates included in the analysis. **Reversed direction of an association.

**References**

1. Thomas F, Renaud F, Benefice E, Meeus T de, Guegan JF. International Variability of Ages at Menarche and Menopause: Patterns and Main Determinants. Hum Biol. 2001;73(2):271–90.

2. Chisholm JS, Quinlivan JA, Petersen RW, Coall DA. Early Stress Predicts Age at Menarche and First Birth, Adult Attachment, and Expected Lifespan. Hum Nat. 2005;16(3):233–65.

3. Danker-Hopfe H. Menarcheal Age In Europe. Yearb Phys Anthropol. 1986;29:81–112.

4. Walker R, Gurven M, Hill K, Migliano A, Chagnon N, De Souza R, et al. Growth rates and life histories in twenty-two small-scale societies. Am J Hum Biol. 2006;18(3):295–311.

5. Kramer KL. Early sexual maturity among Pumé foragers of Venezuela: Fitness implications of teen motherhood. Am J Phys Anthropol. 2008;136(3):338–50.

6. Ellison PT. Morbidity, Mortality, and Menarche. Hum Biol. 1981;53(4):635–43.

7. Jacobsen BK, Heuch I, Kvåle G. Association of low age at menarche with increased all-cause mortality: A 37-year follow-up of 61,319 Norwegian women. Am J Epidemiol. 2007;166(12):1431–7.

8. Jacobsen BK, Oda K, Knutsen SF, Fraser GE. Age at menarche, total mortality and mortality from ischaemic heart disease and stroke: the Adventist Health Study, 1976-88. Int J Epidemiol. 2009;38(1):245–52.

9. Lakshman R, Forouhi NG, Sharp SJ, Luben R, Bingham SA, Khaw K-T, et al. Early Age at Menarche Associated with Cardiovascular Disease and Mortality. J Clin Endocrinol Metab. 2009;94(12):4953–60.

10. Štukovský R, Valšik JA, Bulai-Ştirbu MARY. Family size and menarcheal age in Constanza, Roumania. Hum Biol. 1967;39(3):277–83.

11. Zacharias L, Wurtman RJ. Age at menarche: genetic and environmental influences. N Engl J Med. 1969;280(16):868–75.

12. Milicer H. Age at menarche of girls in Wrocław, Poland, in 1966. Hum Biol. 1968;40(2):249–59.

13. Malina RM. Menarche in athletes: a synthesis and hypothesis. Ann Hum Biol. 1983;10(1):1–24.

14. Morris DH, Jones ME, Schoemaker MJ, Ashworth A, Swerdlow AJ. Determinants of age at menarche in the UK: analyses from the Breakthrough Generations Study. Br J Cancer. 2010;103(11):1760–4.

15. Amir D, Jordan MR, Bribiescas RG. A longitudinal assessment of associations between adolescent environment, adversity perception, and economic status on fertility and age of menarche. PLoS One. 2016;11(6):1–16.

16. Padez C. Social background and age at menarche in Portuguese university students: A note on the secular changes in Portugal. Am J Hum Biol. 2003;15(3):415–27.

17. Padez C. Age at menarche of schoolgirls in Maputo, Mozambique. Ann Hum Biol. 2003;30(4):487–95.

18. Rebacz E. Age at menarche in schoolgirls from Tanzania in light of socioeconomic and sociodemographic conditioning. Coll Antropol. 2009;33(1):23–9.

19. Deardorff J, Abrams B, Ekwaru JP, Rehkopf DH. Early puberty and adolescent pregnancy: the influence of alcohol use. Ann Epidemiol. 2014;24(10):727–33.

20. Wronka I, Pawlińska-Chmara R. Menarcheal age and socio-economic factors in Poland. Ann Hum Biol. 2005;32(5):630–8.

21. Bielicki T, Welon Z. Growth data as indicators of social inequalities: The case of Poland. Am J Phys Anthropol. 1982;25(S3):153–67.

22. Łaska-Mierzejewska T, Milicer H, Piechaczek H. Age at menarche and its secular trend in urban and rural girls in Poland. Ann Hum Biol. 1982;9(3):227–33.

23. Roberts DF, Rozner LM, Swan A V. Age at menarche, physique and environment in industrial North East England. Acta Paediatr. 1971;60(2):158–64.

24. Osteria TS. Nutritional status and menarche in a rural community in the Philippines. Philipp J Nutr. 1983;36(4):150–6.

25. Eveleth PB, Tanner JM. Worldwide variation in human growth. Second Edi. New York, NY 10011, USA: Cambridge University Press.; 1990.

26. Cameron N, Kgamphe JS, Levin Z. Age at menarche and an analysis of secular trends in menarcheal age of South African urban and rural black females. Am J Hum Biol. 1991;3(3):251–5.

27. Chowdhury S, Shahabuddin AKM, Seal AJ, Talukder KK, Hassan Q, Begum RA, et al. Nutritional status and age at menarche in a rural area of Bangladesh. Ann Hum Biol. 2000;27(3):249–56.

28. Rah JH, Shamim AA, Arju UT, Labrique AB, Rashid M, Christian P. Age of onset, nutritional determinants, and seasonal variations in menarche in rural Bangladesh. J Heal Popul Nutr. 2009;27(6):802–7.

29. Song Y, Ma J, Wang H, Wang Z, Hu P, Zhang B, et al. Trends of age at menarche and association with body mass index in Chinese school-aged girls, 1985-2010. J Pediatr. 2014;165(6):1172–1177.e1.

30. Liestøl K. Social conditions and menarcheal age: the importance of early years of life. Ann Hum Biol. 1982;9(6):521–37.

31. Abioye-Kuteyi EA, Ojofeitimi EO, Aina OI, Kio F, Aluko Y, Mosuro O. The Influence of Socioeconomic and Nutritional Status on Menarche in Nigerian School Girls. Nutr Health. 1997;11(3):185–95.

32. Lindgren G. Height, weight and menarche in Swedish urban school children in relation to socio-economic and regional factors. Ann Hum Biol. 1976;3(6):501–28.

33. Berkey CS, Gardner JD, Lindsay Frazier A, Colditz GA. Relation of Childhood Diet and Body Size to Menarche and Adolescent Growth in Girls. Am J Epidemiol. 2000;152(5):446–52.

34. Frisch RE. Population, Food Intake, and Fertility. Science. 1978;199(4324):22–30.

35. Meyer F, Moisan J, Marcoux D, Bouchard C. Dietary and physical determinants of menarche. Epidemiology. 1990;1(5):377–81.

36. Maclure M, Travis LB, Willett W, Macmahon B. A prospective Cohort Study of Nutrient Intake and Age At Menarche. Am J Clin Nutr. 1991;54(4):649–56.

37. Moisan J, Meyer F, Gingras S. Diet and age at menarche. Cancer Causes Control. 1990;1(2):149–54.

38. Moisan J, Meyer F, Gingras S. A nested case-control study of the correlates of early menarche. Am J Epidemiol. 1990;132(5):953–61.

39. Khan AD, Schroeder DG, Martorell R, Haas JD, Rivera J. Early childhood determinants of age at menarche in rural Guatemala. Am J Hum Biol. 1996;8(6):717–23.

40. Merzenich H, Boeing H, Wahrendorf J. Dietary Fat and Sports Activity as Determinants for Age at Menarche. Am J Epidemiol. 1993;138(4):217–24.

41. Sanchez A, Kissinger DG, Phillips RI. A hypothesis on the etiological role of diet on age of menarche. Med Hypotheses. 1981;7(11):1339–45.

42. Carwile J., Willett W., Spiegelman D, Hertzmark E, Rich-Edwards J, Frazier A., et al. Sugar-sweetened beverage consumption and age at menarche in a prospective study of US girls. Hum Reprod. 2015;30(3):675–83.

43. Kralj-Cercek L. The influence of foods, body build, and social origin on the age at menarche. Hum Biol. 1956;28(4):393–406.

44. Kissinger DG, Sanchez A. The association of dietary factors with the age of menarche. Nutr Res. 1987;7(5):471–9.

45. Warren MP. Effects of Undernutrition on Reproductive Function in the Human. Endocr Rev. 1983;4(4):363–77.

46. Satyanarayana K, Naidu AN. Nutrition and menarche in rural Hyderabad. Ann Hum Biol. 1979;6(2):163–5.

47. Freedman DS, Khan LK, Serdula MK, Dietz WH, Srinivasan SR, Berenson GS. Relation of age at menarche to race, time period, and anthropometric dimensions: the Bogalusa Heart Study. Pediatrics. 2002;110(4):e43.

48. Pierce MB, Leon DA. Age at menarche and adult BMI in the Aberdeen children of the 1950s cohort study. Am J Clin Nutr. 2005;82(4):733–9.

49. He Q, Karlberg J. BMI in Childhood and Its Association with Height Gain, Timing of Puberty, and Final Height. Pediatr Res. 2001;49(2):244–51.

50. Wang Y. Is obesity associated with early sexual maturation? A comparison of the association in American boys versus girls. Pediatrics. 2002;110(5):903–10.

51. Simondon KB, Simon I, Simondon F. Nutritional status and age at menarche of Senegalese adolescents. Ann Hum Biol. 1997;24(6):521–32.

52. Kirchengast S, Bauer M. Menarcheal onset is associated with body composition parameters but not with socioeconomic status. Coll Antropol. 2007;31(2):419–25.

53. Ribeiro J, Santos P, Duarte J, Mota J. Association between overweight and early sexual maturation in Portuguese boys and girls. Ann Hum Biol. 2006;33(1):55–63.

54. Jacobson-Dickman E, Lee MM. The influence of endocrine disruptors on pubertal timing. Curr Opin Endocrinol Diabetes Obes. 2009;16(1):25–30.

55. Cooper RL, Stoker TE, Tyrey L, Goldman JM, McElroy WK. Atrazine Disrupts the Hypothalamic Control of Pituitary-Ovarian Function. Toxicol Sci. 2000;53(2):297–307.

56. Kramer KL, Greaves RD. Juvenile Subsistence Effort, Activity Levels, and Growth Patterns: Middle Childhood among Pumé Foragers. Hum Nat. 2011;22(3):303–26.

57. Parker DL. Child labor. The impact of economic exploitation on the health and welfare of children. Minn Med. 1997;80:10 12-3 52-5.

58. Glynn JR, Kayuni N, Floyd S, Banda E, Francis-Chizororo M, Tanton C, et al. Age at menarche, schooling, and sexual debut in Northern Malawi. Cushing B, editor. PLoS One. 2010;5(12):e15334.

59. Sommer M. Menarche: a missing indicator in population health from low-income countries. Public Health Rep. 2013;128(5):399–401.

60. Adadevoh SWK, Agble TK, Hobbs C, Elkins TE. Menarcheal age in Ghanaian school girls. Int J Gynecol Obstet. 1989;30(1):63–8.

61. Low BS, Hazel A, Parker N, Welch KB. Influences on women’s reproductive lives: Unexpected ecological underpinnings. Cross-Cultural Res. 2008;42(3):201–19.
