# Supplementary material for: Is the timing of menarche correlated with mortality and fertility rates?
Source: PLoS One. 2019 Apr 18;14(4):e0215462. doi: 10.1371/journal.pone.0215462 (PMC6472797; doi:10.1371/journal.pone.0215462)
Supplement: S2 Table — (DOCX) [file pone.0215462.s002.docx]

**S2 Table. Mean age at menarche for 89 countries with year, source population, and reference.**

| Country | Year | Mean age at menarche | Source population | Reference |
| --- | --- | --- | --- | --- |
| Albania | 2003 | 14,4** | data collected from ovarian cancer patients (cases and controls) | [2] |
| Algeria | 1993 | 14,3 | data collected in Mustafa Hospital of Algiers | [1] |
| Argentina | 1995 | 12,59 | students from Cordoba National University | [1] |
| Australia | 1972 | 12,65 | Sydney metropolitan area | [4] |
| Bangladesh | 1993 | 15,8 | Matlab | [1] |
| Belgium | 1980 | 13,2* | Flemish schoolgirls | [5] |
| Bolivia | 1985 | 13,4 | Aymara | [7] |
| Brazil | 1978 | 12,5* | Sao Paulo (rich, poor) | [7] |
| Bulgaria | 1997 | 11,96 | Sofia | [6] |
| Cameroon | 1999 | 13,81* | Urban (Yaoundé), suburban, rural | [11] |
| Canada | 2010 | 12,72 | *Nationally representative* | [8] |
| Chile | 2004 | 12,7 | Santiago de Chile | [9] |
| China | 2010 | 12,47* | *Nationally representative* | [10] |
| Colombia | 1991 | 12,8 | 11 institutions in Colombian cities | [1] |
| Congo (Brazza) | 1982 | 12 | - | [1] |
| Congo (Democratic republic) | 1984 | 13,83 | Kinshasa | [1] |
| Croatia | 2010 | 12,31 | Zagreb | [22] |
| Cuba | 1980 | 13,1 | *Nationally representative* | [1] |
| Denmark | 1998 | 13 | Northern Zealand region | [1] |
| Dominican Republic | 1990 | 12,6 | nine regions of the Dominican Republic | [1] |
| Ecuador | 2005 | 14 | Guayaquil | [13] |
| Egypt, Arab Rep. | 2005 | 13 | *Nationally representative* | [14] |
| Estonia | 1999 | 12,8 | *Nationally representative* | [15] |
| Ethiopia | 2007 | 15,8 | towns of Dabat and Kola Diba, Northwest Ethiopia | [16] |
| Fiji | 1985 | 13,6 | Melanesians | [7] |
| Finland | 1984 | 13,2 | 5 cities and 12 rural communities | [1] |
| France | 2010 | 12,8 | *Nationally representative* | [17] |
| Gambia, The | 2008 | 14,9 | rural Gambia | [20] |
| Georgia | 1979 | 12,5 | Tbilisi | [7] |
| Germany | 1989 | 13.1 | Bremerhaven, North Germany | [12] |
| Ghana | 1986 | 13,98 | Kumasi district, Ashanti region | [19] |
| Greece | 2006 | 12,29 | Greater Athens area | [21] |
| Guatemala | 1995 | 13,75 | Four Guatemalan villages | [1] |
| Haiti | 1995 | 15,37 | *Nationally representative* | [1] |
| Hungary | 1993 | 12,9 | Pécs | [1] |
| Iceland | 1972 | 13,1 | Reykjavik | [7] |
| India | 1980 | 14,31 | Punjab | [1] |
| Indonesia | 1990 | 13 | Jakarta | [1] |
| Iran, Islamic Rep. | 2002 | 12,91 | Shiraz, Southern Iran | [24] |
| Iraq | 1969 | 13,8* | Baghdad (rich, poor) | [7] |
| Ireland | 2006 | 12,53 | *Nationally representative* | [23] |
| Israel | 1982 | 13,29 | Jerusalem | [1] |
| Italy | 1992 | 12,2 | - | [1] |
| Jamaica | 1988 | 13,1 | *Nationally representative* | [1] |
| Japan | 1980 | 12,4 | Tokyo urban districts | [26] |
| Jordan | 2012 | 12,95* | Amman city | [25] |
| Kenya | 1987 | 14,4 | 4 teacher and 1 agricultural training colleges | [1] |
| Korea, Rep. (Southern Korea) | 1986 | 13,9 | data collected from the university students | [1] |
| Kuwait | 2010 | 12,41 | *Nationally representative* | [27] |
| Malawi | 2010 | 15,1 | Karonga District | [31] |
| Malaysia | 1985 | 14,2 | village in Terengganu state | [1] |
| Mali | 1997 | 14,4 | Segou Region | [29] |
| Mexico | 1993 | 12,4 | Metropolitan Area of Mexico City | [1] |
| Morocco | 1987 | 14,26 | Marrakech | [28] |
| Mozambique | 2000 | 13,9 | Maputo | [30] |
| Nepal | 1983 | 16,2 | Upper Chumik, Mustang District, Dhaulagiri Zone | [1] |
| Netherlands | 1997 | 13,15 | *Nationally representative* | [33] |
| New Zealand | 1994 | 12,9 | *Nationally representative* | [1] |
| Nicaragua | 1971 | 14 | data collected in General Hospital of Managua | [1] |
| Nigeria | 1997 | 13,94 | Ile-Ife, Osun State | [32] |
| Norway | 1985 | 13,34 | Oslo | [34] |
| Oman | 1991 | 13,3 | Muscat, Sour, Sohar, Samail and Salalah areas | [35] |
| Papua New Guinea | 1992 | 15,8 | Karimui and Daribi census divisions, Simbu Province | [1] |
| Peru | 1988 | 13,23 | Chiclayo | [1] |
| Philippines | 1988 | 13,6 | *Nationally representative* | [1] |
| Poland | 1982 | 13,06 | Warsaw | [1] |
| Portugal | 2003 | 12,32 | students from the University of Coimbra | [36] |
| Romania | 1967 | 13,47 | Constanza | [37] |
| Russia | 1997 | 13 | Moscow | [1] |
| Saudi Arabia | 2002 | 13,05 | data collected in King Khalid University Hospital, Rijád | [38] |
| Senegal | 1997 | 16,1 | Niakhar region (rural areas) | [1] |
| Singapore | 1968 | 12,7* | Singapore (rich, average, poor) | [7] |
| Somalia | 1975 | 14,78 | Mogadishu, Baidoa, Kisimajo | [1] |
| South Africa | 1991 | 14,03 | Ubombo, KwaZulu | [41] |
| Spain | 1997 | 12,31 | Catalonia | [1] |
| Sri Lanka | 1983 | 13,5 | three districts | [1] |
| Sudan | 1983 | 13,75* | Khartoum | [1] |
| Sweden | 1976 | 13.1 | Stockholm | [1] |
| Switzerland | 1996 | 13 | Geneva | [1] |
| Tanzania | 2005 | 15,9 | town of Mafinga, Iringa District and mission in Dar es Salaam | [39] |
| Thailand | 1997 | 12,3 | Bangkok | [1] |
| Turkey | 1996 | 13,28 | Ankara | [1] |
| Uganda | 2013 | 13,45* | Gulu District, Northern Uganda (post-conflict) | [40] |
| United Arab Emirates | 1999 | 12,68 | Al-Ain city | [3] |
| United Kingdom | 1992 | 12,3** | *Nationally representative* | [18] |
| United States | 1991 | 12,8 | *Nationally representative* | [1] |
| Yemen, Rep. | 1979 | 14,4 | *Nationally representative* | [1] |
| Zambia | 1995 | 14,2 | Copperbelt and Lusaka Central Provinces | [42] |
| Zimbabwe | 1995 | 13,5 | *Nationally representative* | [1] |

*Average from several records. **Average from two time periods (see Material and Methods).

**References**

1. Thomas F, Renaud F, Benefice E, Meeus T de, Guegan JF. International Variability of Ages at Menarche and Menopause: Patterns and Main Determinants. Hum Biol. 2001;73(2):271–90.

2. Pajenga E, Rexha T, Çeliku S, Bejtja G, Pisha M. Hormonal risk factors for ovarian cancer in the Albanian case-control study. Bosn J basic Med Sci. 2013;13(2):89–93.

3. Badrinath P, Ghazal-Aswad S, Parfitt D, Osman N. Cultural and ethnic barriers in conducting research. Factors influencing menarche in the United Arab Emirates. Saudi Med J. 2004;25(11):1626–30.

4. Harper J, Collins JK. The secular trend in the age of menarche in Australian schoolgirls. J Paediatr Child Health. 1972;8(1):44–8.

5. Wellens R, Malina RM, Beunen G, Lefevre J. Age at menarche in Flemish girls: current status and secular change in the 20th century. Ann Hum Biol. 1990;17(2):145–52.

6. Tomova A, Genov N, Kumanov F, Robeva R. Menarche in Bulgarian--secular trend in twenty century. Akush Ginekol (Sofiia). 2009;48(3):10–4.

7. Eveleth PB, Tanner JM. Worldwide variation in human growth. Second Edi. New York, NY 10011, USA: Cambridge University Press.; 1990.

8. Al-Sahab B, Ardern CI, Hamadeh MJ, Tamim H. Age at menarche in Canada: results from the National Longitudinal Survey of Children & Youth. BMC Public Health. 2010;10(1):736.

9. Codner E, Unanue N, Gaete X, Barrera A, Mook-Kanamori D, Bazaes R, et al. Age of pubertal events in Chilean school age girls and its relationship with socioeconomic status and body mass index. Rev Med Chil. 2004;132(7):801–8.

10. Song Y, Ma J, Wang H, Wang Z, Hu P, Zhang B, et al. Trends of age at menarche and association with body mass index in Chinese school-aged girls, 1985-2010. J Pediatr. 2014;165(6):1172–1177.e1.

11. Pasquet P, Manguelle-Dicoum Biyong A, Rikong-Adie H, Befidi-Mengue R, Garba M-T, Froment A. Age at menarche and urbanization in Cameroon: current status and secular trends. Ann Hum Biol. 1999;26(1):89–97.

12. Ostersehlt D, Danker-Hopfe H. Changes in age at menarche in Germany: Evidence for a continuing decline. Am J Hum Biol. 1991;3(6):647–54.

13. Chedraui PA, Hidalgo LA, Chávez MJ, Miguel GS. Determinant factors in Ecuador related to pregnancy among adolescents aged 15 or less. J Perinat Med. 2005;32(4):337–41.

14. Torres-Mejía G, Cupul-Uicab LA, Allen B, Galal O, Salazar-Martínez E, Lazcano-Ponce EC. Comparative study of correlates of early age at menarche among Mexican and Egyptian adolescents. Am J Hum Biol. 2005;17(5):654–8.

15. Papp K, Part K, Torik S. Noorsoouuring KISS.[The Youth Sexual Maturation Survey KISS.]. Tartu: Estonian Family Planning Association. 2001.

16. Zegeye DT, Megabiaw B, Mulu A. Age at menarche and the menstrual pattern of secondary school adolescents in northwest Ethiopia. BMC Womens Health. 2009;9(1):29.

17. Gaudineau A, Ehlinger V, Vayssiere C, Jouret B, Arnaud C, Godeau E. Factors associated with early menarche: results from the French Health Behaviour in School-aged Children (HBSC) study. BMC Public Health. 2010;10(1):175.

18. Morris DH, Jones ME, Schoemaker MJ, Ashworth A, Swerdlow AJ. Secular trends in age at menarche in women in the UK born 1908-93: Results from the breakthrough generations study. Paediatr Perinat Epidemiol. 2011;25(4):394–400.

19. Adadevoh SWK, Agble TK, Hobbs C, Elkins TE. Menarcheal age in Ghanaian school girls. Int J Gynecol Obstet. 1989;30(1):63–8.

20. Prentice S, Fulford AJ, Jarjou LMA, Goldberg GR, Prentice A. Evidence for a downward secular trend in age of menarche in a rural Gambian population. Ann Hum Biol. 2010;37(5):717–21.

21. Papadimitriou A, Fytanidis G, Douros K, Bakoula C, Nicolaidou P, Fretzayas A. Age at menarche in contemporary Greek girls: evidence for levelling-off of the secular trend. Acta Paediatr. 2008;97(6):812–5.

22. Veček N, Veček A, Petranović MZ, Tomas Ž, Arch-Veček B, Škarić-Jurić T, et al. Secular trend of menarche in Zagreb (Croatia) adolescents. Eur J Obstet Gynecol Reprod Biol. 2012;160(1):51–4.

23. O’Connell A, Gavin A, Kelly C, Molcho M, Nic GS. The mean age at menarche of Irish girls in 2006. Irish Med J. 2009;102(3):76–9.

24. Ayatollahi SMT, Dowlatabadi E, Ayatollahi SAR. Age at menarche in Iran. Ann Hum Biol. 2002;29(4):355–62.

25. Bata MS. Age at menarche, menstrual patterns, and menstrual characteristics in Jordanian adolescent girls. Int J Gynecol Obstet. 2012;119(3):281–3.

26. Hoshi H, Kouchi M. Secular trend of the age at menarche of Japanese girls with special regard to the secular acceleration of the age at peak height velocity. Hum Biol. 1981;53(4):593–8.

27. Al-Awadhi N, Al-Kandari N, Al-Hasan T, AlMurjan D, Ali S, Al-Taiar A. Age at menarche and its relationship to body mass index among adolescent girls in Kuwait. BMC Public Health. 2013;13(1):29.

28. Boëtsch G, Loukid M, Montero P. Menarcheals age and socio-economical factors in Marrakech (Morocco). Int J Anthropol. 1995;10(4):183–7.

29. Pawloski LR. Growth and development of adolescent girls from the Segou Region of Mali (West Africa). Am J Phys Anthropol. 2002;117(4):364–72.

30. Padez C. Age at menarche of schoolgirls in Maputo, Mozambique. Ann Hum Biol. 2003;30(4):487–95.

31. Glynn JR, Kayuni N, Floyd S, Banda E, Francis-Chizororo M, Tanton C, et al. Age at menarche, schooling, and sexual debut in Northern Malawi. Cushing B, editor. PLoS One. 2010;5(12):e15334.

32. Abioye-Kuteyi EA, Ojofeitimi EO, Aina OI, Kio F, Aluko Y, Mosuro O. The Influence of Socioeconomic and Nutritional Status on Menarche in Nigerian School Girls. Nutr Health. 1997;11(3):185–95.

33. Keizer-Schrama SMPF d. M, Mul D. Trends in pubertal development in Europe. Apmis. 2001;109(S103):S164–70.

34. Liestøl K, Rosenberg M. Height, weight and menarcheal age of schoolgirls in Oslo - an update. Ann Hum Biol. 1995;22(3):199–205.

35. Musaiger AO. Height, weight and menarcheal age of adolescent girls in Oman. Ann Hum Biol. 1991;18(1):71–4.

36. Padez C. Social background and age at menarche in Portuguese university students: A note on the secular changes in Portugal. Am J Hum Biol. 2003;15(3):415–27.

37. Štukovský R, Valšik JA, Bulai-Ştirbu MARY. Family size and menarcheal age in Constanza, Roumania. Hum Biol. 1967;39(3):277–83.

38. Babay ZA, Addar MH, Shahid K, Meriki N. Age at menarche and the reproductive performance of Saudi women. Ann Saudi Med. 2004;24(5):354–6.

39. Rebacz E. Age at menarche in schoolgirls from Tanzania in light of socioeconomic and sociodemographic conditioning. Coll Antropol. 2009;33(1):23–9.

40. Mpora BO, Piloya T, Awor S, Ngwiri T, Laigong P, Mworozi EA, et al. Age at menarche in relation to nutritional status and critical life events among rural and urban secondary school girls in post-conflict Northern Uganda. BMC Womens Health. 2014;14(1):66.

41. Cameron N, Kgamphe JS, Levin Z. Age at menarche and an analysis of secular trends in menarcheal age of South African urban and rural black females. Am J Hum Biol. 1991;3(3):251–5.

42. Pillai VK. Age at menarche among adolescent females in Zambia: implication for family formation. Int J Sociol Fam. 1995;25:33–8.
