# Supplementary material for: Is the timing of menarche correlated with mortality and fertility rates?
Source: PLoS One. 2019 Apr 18;14(4):e0215462. doi: 10.1371/journal.pone.0215462 (PMC6472797; doi:10.1371/journal.pone.0215462)
Supplement: S3 Table — (DOCX) [file pone.0215462.s003.docx]

**S3 Table. Univariate statistics for non-normally distributed data with inter-quartile range (IQR), median, min and max values for the covariates included in the analyses.**

| **Covariate** | **N** | **IQR** | **Median** | **Min** | **Max** |
| --- | --- | --- | --- | --- | --- |
| Mean age at menarche | 89 | 1.28 | 13.28 | 11.96 | 16.2 |
| Life expectancy at birth | 89 | 15.45 | 70.2 | 47.3 | 81.2 |
| Fertility rate | 89 | 3.50 | 3.12 | 1.19 | 8.84 |
| Adolescent fertility (15-19) | 89 | 87.91 | 60.59 | 3.6 | 191 |
| Maternal mortality ratio | 73 | 471 | 60 | 2 | 1100 |
| Infant mortality rate | 87 | 53.5 | 26.8 | 3.5 | 147.7 |
| Under 5 mortality rate | 89 | 85.55 | 33.9 | 4.2 | 226.2 |
| Adult female mortality rate | 87 | 186.36 | 131.09 | 45.86 | 594.63 |
| Adult male mortality rate | 87 | 180.27 | 225.70 | 85.40 | 612.98 |
| Total adult mortality rate | 87 | 177.77 | 179.06 | 69.02 | 593.71 |
| Average size of household | 70 | 2.11 | 3.78 | 2.2 | 8.14 |
| Energy use (kg of oil) | 79 | 3009.82 | 1429.97 | 86.56 | 11306.78 |
| Fossil fuel energy consumption (%) | 78 | 48.36 | 75.99 | 0 | 100 |
| Rural population (%) | 89 | 40.57 | 44.77 | 0 | 93.13 |
| Gross domestic product (GDP) per capita | 84 | 12366.37 | 2240.88 | 143.59 | 32855.31 |
| Livestock production index | 89 | 35.36 | 85.26 | 26.99 | 526.07 |
| Food production index | 89 | 32.49 | 87.39 | 31.55 | 516.46 |
| Energy consumption per capita (kcal/person/day) | 72 | 1087.5 | 2895 | 1753 | 3700 |
| Sugar consumption (g/person/day) | 75 | 65.76 | 84.93 | 2.74 | 172.6 |
| Body mass index (BMI) female | 79 | 3.01 | 24.78 | 18.76 | 31.16 |
| Out of primary school, female | 56 | 326920.25 | 70595 | 24 | 4171370 |
| Primary completion rate, female (%) | 55 | 39.45 | 87.04 | 12.56 | 106.81 |
