# Supplementary material for: Is the timing of menarche correlated with mortality and fertility rates?
Source: PLoS One. 2019 Apr 18;14(4):e0215462. doi: 10.1371/journal.pone.0215462 (PMC6472797; doi:10.1371/journal.pone.0215462)
Supplement: S4 Table — (DOCX) [file pone.0215462.s004.docx]

**S4 Table. Analysis of spatial autocorrelation (Mantel’s coefficient) between mean age at menarche and the remaining covariates.**

| **Covariate** | **N** | **Mantel’s coefficient** | **p-value** |
| --- | --- | --- | --- |
| **Demographic** | | | |
| Mean age at menarche | 89 | -0.012 | 0.551 |
| Life expectancy at birth | 89 | -0.021 | 0.653 |
| Fertility rate | 89 | -0.045 | 0.857 |
| Adolescent fertility (15-19) | 89 | -0.001 | 0.460 |
| Maternal mortality ratio | 73 | -0.042 | 0.727 |
| Infant mortality | 87 | -0.028 | 0.687 |
| Under-five mortality | 89 | -0.052 | 0.839 |
| Adult female mortality | 87 | -0.041 | 0.752 |
| Adult male mortality | 87 | -0.020 | 0.610 |
| Total adult mortality | 87 | -0.032 | 0.708 |
| **Socioeconomic** | | | |
| Average size of household | 70 | -0.041 | 0.701 |
| Rural population | 88 | 0.061 | 0.054 |
| Energy use | 78 | 0.043 | 0.245 |
| Fossil fuel energy consumption | 78 | -0.077 | 0.969 |
| Gross domestic product (GDP) per capita | 84 | 0.001 | 0.462 |
| Livestock production index | 89 | 0.046 | 0.281 |
| Food production index | 89 | 0.041 | 0.293 |
| **Nutritional** | | | |
| Energy consumption per capita | 72 | 0.036 | 0.175 |
| Sugar consumption | 75 | 0.012 | 0.058 |
| Body mass index (BMI) female | 79 | 0.010 | 0.415 |
| **Educational** | | | |
| Out of primary school female | 56 | -0.086 | 0.820 |
| Primary completion rate female | 55 | -0.085 | 0.821 |
