# Supplementary material for: Is the timing of menarche correlated with mortality and fertility rates?
Source: PLoS One. 2019 Apr 18;14(4):e0215462. doi: 10.1371/journal.pone.0215462 (PMC6472797; doi:10.1371/journal.pone.0215462)
Supplement: S5 Table — (DOCX) [file pone.0215462.s005.docx]

**S5 Table. Rho-Spearman correlation of the tested covariates with mean age at menarche.**

| **Covariate** | **N** | **Spearman’s coefficient** | **p-value** |
| --- | --- | --- | --- |
| **Demographic** | | | |
| Life expectancy at birth | 89 | -0.681 | 0.001 |
| Fertility rate | 89 | 0.681 | 0.001 |
| Adolescent fertility (15-19) | 89 | 0.536 | 0.001 |
| Maternal mortality ratio | 89 | 0.649 | 0.001 |
| Infant mortality | 73 | 0.646 | 0.001 |
| Under-five mortality | 87 | 0.674 | 0.001 |
| Adult female mortality | 89 | 0.685 | 0.001 |
| Adult male mortality | 87 | 0.579 | 0.001 |
| Total adult mortality | 87 | 0.643 | 0.001 |
| **Socioeconomic** | | | |
| Average size of household | 70 | 0.453 | 0.001 |
| Rural population | 89 | 0.652 | 0.001 |
| Energy use | 78 | -0.600 | 0.001 |
| Fossil fuel energy consumption | 78 | -0.475 | 0.001 |
| Gross domestic product (GDP) per capita | 84 | -0.659 | 0.001 |
| Livestock production index | 89 | -0.309 | 0.003 |
| Food production index | 89 | -0.367 | 0.001 |
| **Nutritional** | | | |
| Energy consumption per capita | 72 | -0.627 | 0.001 |
| Sugar consumption | 75 | -0.550 | 0.001 |
| Body mass index (BMI) female | 79 | -0.475 | 0.001 |
| **Educational** | | | |
| Out of primary school female | 56 | 0.596 | 0.001 |
| Primary completion rate female | 55 | -0.532 | 0.001 |
